# Infection with Possible Novel Parapoxvirus in Horse, Finland, 2013

**DOI:** 10.3201/eid2207.151636

**Published:** 2016-07

**Authors:** Niina Airas, Maria Hautaniemi, Pernilla Syrjä, Anna Knuuttila, Niina Putkuri, Lesley Coulter, Colin J. McInnes, Olli Vapalahti, Anita Huovilainen, Paula M. Kinnunen

**Affiliations:** University of Helsinki Faculty of Veterinary Medicine, Helsinki, Finland (N. Airas, P. Syrjä, A. Knuuttila, O. Vapalahti, P.M. Kinnunen);; Finnish Food Safety Authority Evira, Helsinki (M. Hautaniemi, A. Huovilainen);; Haartman Institute, University of Helsinki, Helsinki (N. Putkuri, O. Vapalahti, P.M. Kinnunen);; Moredun Research Institute, Penicuik, UK (L. Coulter, C.J. McInnes);; Helsinki University Central Hospital, Helsinki (O. Vapalahti)

**Keywords:** Clinical, dermatitis, granuloma, equine, horse, viruses, poxvirus, parapoxvirus, PCR, phylogeny, sequence, zoonosis, Finland

## Abstract

A horse in Finland exhibited generalized granulomatous inflammation and severe proliferative dermatitis. After euthanization, we detected poxvirus DNA from a skin lesion sample. The virus sequence grouped with parapoxviruses, closely resembling a novel poxvirus detected in humans in the United States after horse contact. Our findings indicate horses may be a reservoir for zoonotic parapoxvirus.

Parapoxviruses (PPVs) are zoonotic viruses that have been known for centuries to cause contagious pustular skin infections in sheep, goats, and cattle worldwide. These viruses also infect other animals, such as red deer, seals, camels, reindeer, and domestic cats (*1*,*2*). In the genus *Parapoxvirus*, 4 species are currently recognized: Orf virus (ORFV), bovine papular stomatitis virus (BPSV), pseudocowpox virus (PCPV), and parapoxvirus of red deer in New Zealand (PVNZ) (*3*). In Finland, ORFV has repeatedly been detected in sheep, PCPV in cattle, and ORFV and PCPV in reindeer and humans (*4*,*5*). PPVs replicate in epidermal keratinocytes and generally produce pustular lesions at the infection site, which is typically around the mouth, tongue, lips, or teats of mammals. Primary lesions can be severe and proliferative but in uncomplicated cases scab within 1 week and resolve in 4–6 weeks. If the disease is complicated by secondary bacteria, the lesions can become ulcerative and necrotic, delaying healing (*6*). 

All recognized PPV species except PVNZ have been identified in humans. Manifestations of human PPV infections (“farmyard pox”) are typically seen on the hands of persons who had contact with infected ruminants. Recently, Osadebe et al. (*7*) reported novel poxvirus infections in 2 humans who had contact with domestic animals including horses and donkeys.

In Finland, PPV infections are common in ruminants, but unknown in horses; 3.1% of horses are seropositive for orthopoxviruses (OPV), but such infections appear to be subclinical (*8*). We describe a severe disease including dermatitis in a horse and identification of possible novel zoonotic parapoxvirus from a skin lesion.

## The Patient

**Figure 1:**
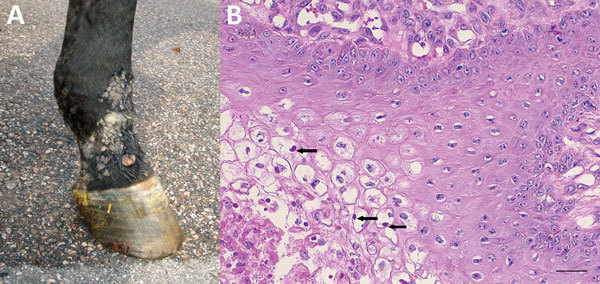
Macroscopic and histologic images of horse infected with possible novel parapoxvirus, Finland, 2013. A) Proliferative and ulcerative skin lesions were seen multifocally on the muzzle, ventral abdomen, and lower limbs (pictured). B) The main histological changes in samples of the skin lesions were severe multifocal lymphohistiocytic dermatitis with marked ballooning degeneration of the stratum granulosum and eosinophilic intrasytoplasmic inclusion bodies in many keratinocytes (arrows). Scale bar indicates 50 μm.

A rapidly progressive disease developed in a 2-year-old Standardbred stallion in Finland; clinical signs were fever, scrotal swelling, and ventral edema (Technical Appendix Figure); multifocal, hard, nodular skin lesions (Figure 1, panel A) and moderately enlarged lymph nodes were also apparent. The horse was apathetic and lost weight despite a good appetite. The attending clinicians suspected generalized lymphoma. However, a biopsy sample taken from nodular skin lesions showed proliferative dermatitis (Table). The horse had secondary immune-mediated hemolytic anemia 1.5 months after onset of disease; because the prognosis was poor, the horse was euthanized in September 2013. The body was received at the University of Helsinki Faculty of Veterinary Medicine (Helsinki, Finland) for a postmortem examination that month.

**Table:** Histopathologic and PCR findings in samples from a horse infected with parapoxvirus, Finland, 2013*

Source	Histopathology	PPV PCR(*10*), RNA polymerase gene	Pan-PPV PCR (*11*), high GC, ENV gene	Other poxvirus PCRs (*6**,**10*)†
Skin lesions	Severe multifocal proliferative lymphohistiocytic dermatitis	Positive	Positive	Negative
Postmortem samples				
Skin lesions	Severe multifocal proliferative lymphohistiocytic dermatitis	Positive	Positive	Negative
Lung	Severe diffuse lymphohistiocytic interstitial pneumonia	Negative	Negative	Negative
Intestines	Moderate diffuse lymphohistiocytic enteritis	Negative	Negative	Negative
Intestinal lymph nodes	Moderate multifocal lymphohistiocytic inflammation	Negative	Negative	Negative

*ENV, envelope phospholipase; GC, guanine-cytosine; PPV, parapoxvirus. †See Technical Appendix Table.

In necropsy, the horse was found to be thin and poorly muscled. Multifocal, nodular, dry, hard, proliferative lesions in the skin were mainly on the muzzle, lower forelimbs, and ventral abdomen. Moderate edema was present in the abdomen, scrotum, and all limbs. Thickened and hyperemic mucosa in the small intestine, moderately swollen mesenteric lymph nodes, and ascites were visible.

Histologically, the skin lesions were characterized by severe multifocal lymphohistiocytic dermatitis with intraepidermal vesicles caused by marked ballooning degeneration of the stratum granulosum (Figure 1, panel B). Eosinophilic intracytoplasmic inclusion bodies were seen in keratinocytes. Intestinal tissue, lungs, and mesenteric lymph nodes showed chronic, lymphohistiocytic inflammatory changes (Table). Special stains for mycobacteria were negative.

Because the histological findings of the skin samples suggested poxvirus infection, we collected a frozen plain skin sample and slices from formalin-fixed, paraffin-embedded skin, lung, lymph node, and spleen for virological studies. We attempted virus isolation from the skin sample in green monkey and baby hamster kidney cells and saw negative results. DNA was extracted by using the DNeasy Blood & Tissue Kit (QIAGEN, Hilden, Germany), but no OPV DNA was detectable by real-time PCR (*9*) (Technical Appendix Table). However, PPV DNA or that of a closely related virus was present in the skin samples: both the Pan-PPV PCR targeting the PPV envelope phospholipase gene (ENV) (*11*) and the high-GC (guanine-cytosine) pan-pox PCR targeting the large subunit of the poxvirus RNA polymerase gene (RPO147) (*10*) produced amplicons (Table), although several other primer pairs targeting PPV genes were negative (Technical Appendix Table).

**Figure 2:**
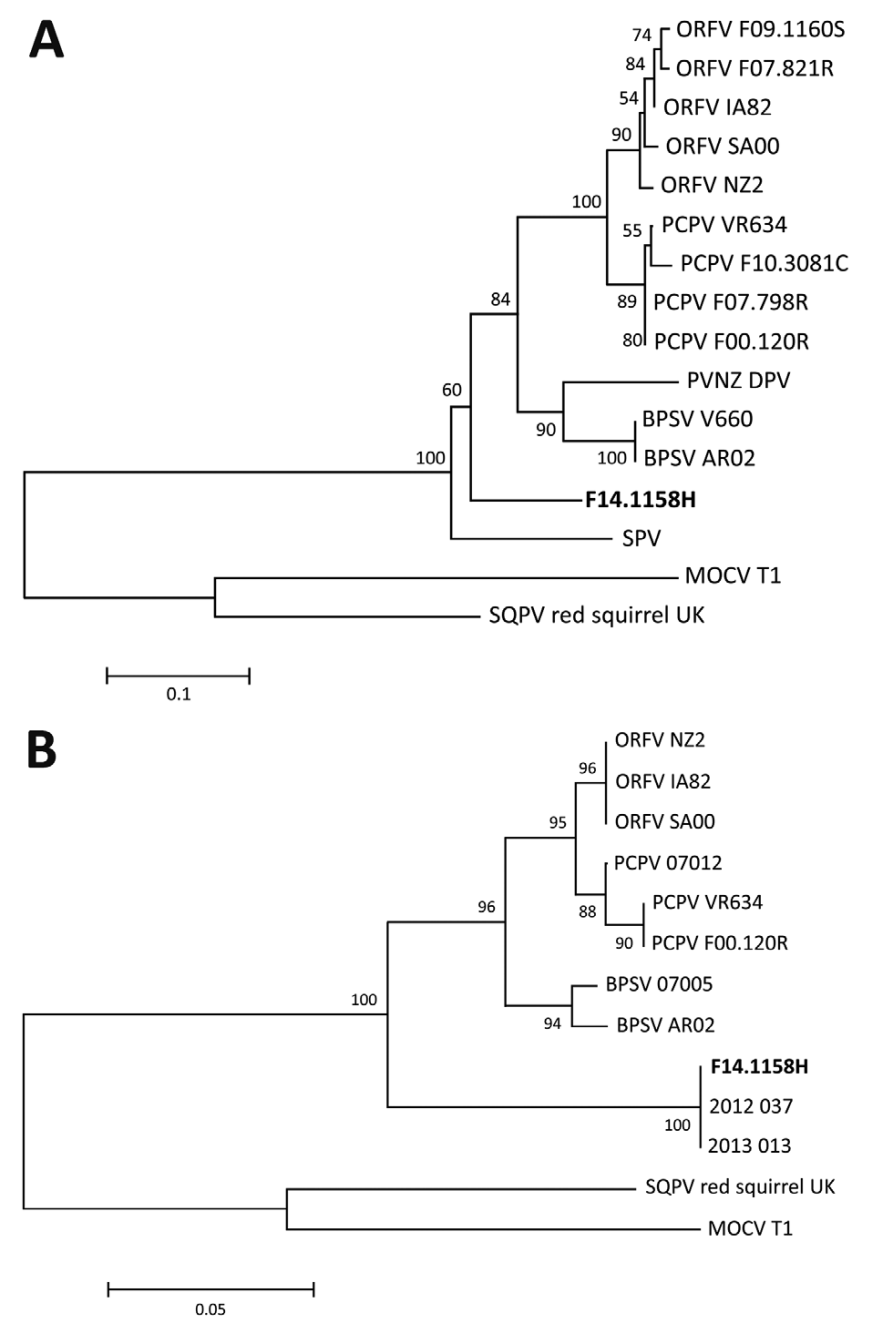
Phylogenetic analyses of sequences amplified from skin lesion of horse infected with possible novel parapoxvirus, Finland, 2013 (poxvirus variant F14.1158H), and other poxviruses. Trees were generated by using the neighbor-joining method in MEGA 6 software (http://www.megasoftware.net) (*12*), based on A) 184 aa of envelope phospholipase gene and B) 195 aa of viral RNA polymerase gene RP0147. GenBank accession numbers for sequences used in the analyses: JF773701 (Orf virus [ORFV] F07.821R), JF773703 (ORFV F09.1160S), AY386263 (ORFV IA82), AY386264 (ORFV SA00), DQ184476 (ORFV NZ2), GQ329670 (pseudocowpox virus [PCPV] VR634), JF773695 (PCPV F10.3081C), JF773692 (PCPV F07.798R), GQ329669 (PCPV F00.120R), AY453655 (parapoxvirus of red deer in New Zealand [PVNZ] DPV), AY453664 (bovine papular stomatitis virus [BPSV] V660), AY386265 (BPSV AR02), AF414182 (sealpoxvirus [SPV]), U60315 (molluscum contagiosum virus [MOCV] subtype 1), HE601899 (squirrelpox virus [SQPV] red squirrel UK), GQ902051.1 (PCPV 07012), GQ902054.1 (BPSV 07005), KM491712 (2013_013), and KM491713 (2012_037). The final 2 sequences originated from recent cases in humans with equine contacts in the United States (*7*). The reliability of the trees was determined by 1,000 dataset bootstrap resampling; the percentage of replicate trees in which the associated taxa clustered together is shown in the branches. Scale bars indicate amino acid substitutions per site.

Sequencing of the PCR products showed that the ENV (GenBank accession no. KR863114) and RPO147 (GenBank accession no. KR827441) sequences shared 80%–89% nt and aa identity with other PPVs, depending on the virus species. The RPO147 sequence was 99%–100% identical at nt level and 100% identical at aa level to the sequences of the 2 recent poxvirus isolates (2012_37 and 2013_013 RPO147) from humans in the United States (*7*). In phylogenetic analyses, the sequences from the horse in this study and from these human patients grouped together, forming a different lineage within the PPVs and separate from other related poxviruses, molluscum contagiosum virus and squirrelpox virus (Figure 2). The equine poxvirus was designated F14.1158H.

Although the skin lesions showed poxvirus infection, formalin-fixed samples from internal organs contained no viral inclusion bodies and were negative for PPV by PCR. This finding is in accordance with the fact that PPVs are specialized to replicate in the highly specific immune environment of skin (*13*). Further investigations are required to show whether the poxvirus caused the generalized infection in addition to dermatitis.

The owner, breeder, and trainers of the horses on the farm where this horse became ill were unaware of any other animal or zoonotic cases in the premises and disclosed no contact between the horse and ruminants. The horse had lived in contact with many horses and several dogs and cats in 3 locations in southern parts of western and eastern Finland before being transferred to the last training stable. A few months before onset of clinical signs, the horse had been trained at a farm where cows had been kept 25 years earlier. During the illness, the horse lived in a stable with 17 horses, shared corrals and equipment, and had muzzle contact with 2 horses in adjacent stalls. Despite the direct and indirect contacts, all other horses, the 3 caretakers, and the trainer remained asymptomatic.

## Conclusions

We report a clinical equine infection with a novel poxvirus in Finland. The infection is at least of dermatitic relevance for horses, and veterinary awareness is needed. The sequence analysis based on conserved genes revealed a close relationship between this isolate and recent poxvirus isolates from humans with horse contact in the United States (*7*). Although sequence data are limited and the geographic distance between this equine case and the recent cases in humans is remote, the close genetic relatedness suggests that horses have a possible role as reservoir or vector of an emerging zoonotic poxvirus, necessitating medical awareness and emphasizing the importance of the One Health approach (https://www.onehealthcommission.org/). The horse as an origin for zoonoses is not uncommon: as many as 58% of emerging zoonotic pathogens infect ungulates (*14*). As for cowpox virus, horse and human may be infected from a common source, such as rodents, and not necessarily from each other. This case appeared sporadic and not very contagious, and the transmission route remained unresolved. Further studies are needed to elucidate ecology, epidemiology, prevalence, and possible zoonotic transmission.

As our limited sequence analysis suggests, the virus we detected is most closely related to PPVs and may merit being classified as a new *Parapoxvirus* species. However, many of the established PPV primer pairs did not produce PCR product, which suggests that the virus is different from the established PPV species and may represent a new poxvirus genus. More sequence data are needed to validate the taxonomic classification of the equine poxvirus. In conclusion, our results provide further evidence that horses are a possible source of the new poxvirus infection recently observed in humans.

**Technical Appendix.** Summary of PCR results on a sample from a horse infected with possible novel parapoxvirus in Finland, 2013, and image of the horse.
