# Supplementary material for: Infection with Possible Novel Parapoxvirus in Horse, Finland, 2013
Source: Emerg Infect Dis. 2016 Jul;22(7):1242–5. doi: 10.3201/eid2207.151636 (PMC4918186; doi:10.3201/eid2207.151636)
Supplement: Supplementary file 1 — Technical Appendix. Summary of PCR results on a sample from a horse infected with possible novel parapoxvirus in Finland, 2013, and image of the horse. [file 15-1636-Techapp-s1.pdf]

Article DOI: http://dx.doi.org/10.3201/eid.2207.151636

Infection with Possible Novel Parapoxvirus in Horse, Finland, 2013

Technical Appendix

Technical Appendix Table. Summary of PCR results on a sample from a horse, Finland, 2013*

Virus target	Gene target	Oligos	Oligo sequence, 5'→3'	Result	Comments	Reference
Primers that gave a product:						
PPVs	VACV-Cop F13L (envelope phospholipase)	PPP-1	5′-GTCGTCCACGATGAGCAGCT-3′	Product shows similarity to parapoxviruses	Amplifies all known parapoxviruses	(*1*)
		PPP-4	5′-TACGTGGGAAGCGCCTCGCT-3′			
Pan-poxvirus PCR, high GC content	VACV-Cop J6R (RNA polymerase)	Pan-pox high-GC For	5′-catccccaaggagaccaacgag-3′	Product shows similarity to parapoxviruses	Amplifies poxvirus genomes with G+C content >60%, including the parapoxviruses, molluscum contagiosum virus and crocodilepox virus	(*2*)
		Pan-pox high-GC Rev	5′-TCCTCGTCGCCGTCGAAGTC-3′			
Primers that did not give a product:						
Orthopoxviruses	VACV-Cop-A56R (Hemagglutinin)	PoxHA1.1	5′-GTGATGATGCAACTCTATCATG-3′	No product	Amplifies all orthopoxviruses including cowpox virus, vaccinia virus, and smallpox virus	(*3*)
		PoxHA1.2	5′-TGTAACTAGATCATCGTATGGAGA-3′	No product	Amplifies all orthopoxviruses including cowpox virus, vaccinia virus, and smallpox virus	(*3*)
		Pox FL (anchor probe)	5′-CTAAAAGAATAATGGAATTGGGCTCC-f-3′	No product	Amplifies all orthopoxviruses including cowpox virus, vaccinia virus, and smallpox virus	(*3*)
		Pox Red640 (sensor probe)	5′-LCRed640-ATACCAAGCACTCATAACAACATAATCATTTATATTAT-p-3′	No product	Amplifies all orthopoxviruses including cowpox virus, vaccinia virus, and smallpox virus	(*3*)
ChPV low GC content	VACV-Cop G1L (metalloprotease gene)	Pan-pox low-GC For	5′-ACACCAAAAACTCATATAACTTCT-3′	No product	Amplifies all orthopoxviruses including	(*2*)
		Pan-pox low-GC Rev	5′-CCTATTTTACTCCTTAGTAAATGAT-3′			

Virus target	Gene target	Oligos	Oligo sequence, 5’→3’	Result	Comments	Reference
					cowpox virus, vaccinia virus, and smallpox virus	
ORFV	ORFV117, GIF gene (GM-CSF inhibitory factor gene)	GIFF1<br>GIFR1	5′-TCAGAGTGTTCCTGGCGGTGCTC-3′<br>5′-GTAGAACGTGCTGGAGAAACT-3′	No product	Amplifies products from orf virus and pseudocowpox virus	(*4*)
BPSV	BPSV117, GIF gene (GM-CSF inhibitory factor gene)	BPSGIF-5′<br>BPSGIF-3′	5′-ACACGCCATGCAGCGTGCGCTGCGC-3′<br>5′-GGATTATCACTGTCCGGTGGTCATC-3′	No product	Amplifies bovine papular stomatitis virus	(*4*)
PCPV	PCPV001, 001.3 (IL-10 ortholog)	PCPV-5′<br>PCPV-3′	5′-GGTACACCGGCGAGAGCA-3′<br>5′-CATGGACCGGACGTAAAAGA-3′	No product	Amplifies region specific to pseudocowpox virus containing IL-10 ortholog gene	This report
ChPV	VACV-Cop A3L (major core protein)	A3LFor1<br>A3LRev3	5′-CNTCHACNMABRAYTGG-3′<br>5′-TGYTCYTCRTCNGHCAT-3′	No product	Degenerate primers, had previously amplified a product from a Spanish red squirrel poxvirus, distinct from the UK SQPV	(*5*)
ChPV[†]	VACV-Cop F10L (serine protein kinase)	F10LF958<br>F10LR1167	5′-GAYYTNAARCCNGAYAA-3′<br>5′-AARTGRAARTCRTARWACCA-3′	No product	Degenerate primers, had previously amplified a product from a Canadian red squirrel poxvirus, distinct from the UK SQPV	(*6*)
OPV, CPV<br>ChPV[†]	VACV-Cop F10L (serine protein kinase)	F10LF296<br>F10LR1167	5′-GGAGGATATGGTATAGT<br>5′-AARTGRAARTCRTARWACCA-3′	No product	Had previously amplified a product from a Canadian red squirrel poxvirus, distinct from the UK SQPV	(*6*)
PPV, LPV	VACV-Cop F9L, F10L (intergenic region between serine protein kinase and lipid membrane protein gene)	CanSPVF9/10 For<br>CanSPVF9/10 Rev	5′-TTBAGGATCTGYAMCAGGATGT-3′<br>5′-GGTAYTACGAYTTYCACTTCTT-3′	No product	Degenerate primers, had previously amplified a product from a Spanish red squirrel poxvirus, distinct from the UK SQPV	(*5*)
OPV, CPV	VACV-Cop E9L (DNA polymerase)	PanpolFor1<br>PanpolRev1	5′-AARTTTCCTTCYGTWTTT-3′<br>5′-ATAGAATCYAAYTTRTA-3′	No product	Degenerate primers	This report

*PPV, parapoxvirus; ChPV, *Chordopoxvirinae*; ORFV, orf virus; BPSV, bovine papular stomatitis virus; PCPV, pseudocowpox virus; OPV, orthopoxvirus; CPV, capripoxvirus; LPV, leporipoxvirus.

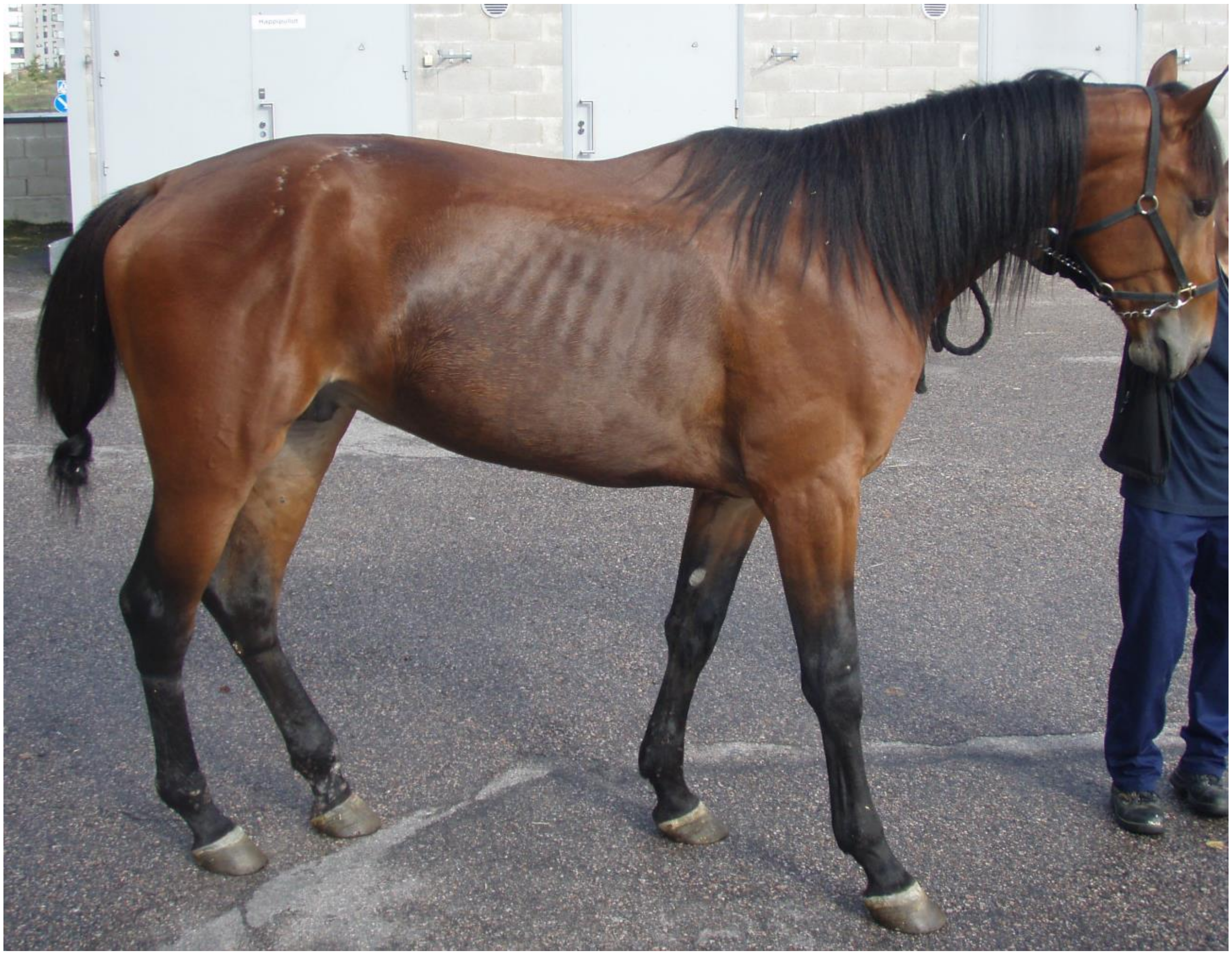

Technical Appendix Figure. A horse infected with a possible novel parapoxvirus in Finland, 2013, showed chronic weight loss, scrotal and ventral edema, and proliferative dermatitis.

References

1. Inoshima Y, Morooka A, Sentsui H. Detection and diagnosis of parapoxvirus by the polymerase chain reaction. J Virol Methods. 2000;84:201–8. http://dx.doi.org/10.1016/S0166-0934(99)00144-5

2. Li Y, Meyer H, Zhao H, Damon IK. GC content-based pan-pox universal PCR assays for poxvirus detection. J Clin Microbiol. 2010;48:268–76. http://dx.doi.org/10.1128/JCM.01697-09

3. Putkuri N, Piiparinen H, Vaheri A, Vapalahti O. Detection of human orthopoxvirus infections and differentiation of smallpox virus with real-time PCR. J Med Virol. 2009;81:146–52. http://dx.doi.org/10.1002/jmv.21385

4. Deane D, Ueda N, Wise LM, Wood AR, Percival A, Jepson C, et al. Conservation and variation of the parapoxvirus GM-CSF inhibitory factor (GIF) proteins. J Gen Virol. 2009;90:970–7. http://dx.doi.org/10.1099/vir.0.006692-0

5. Obon E, Juan-Sallés C, McInnes CJ, Everest DJ. Poxvirus identified in a red squirrel (*Sciurus vulgaris*) from Spain. Vet Rec. 2011;168:86. http://dx.doi.org/10.1136/vr.d204

6. Himsworth CGI, McInnes CJ, Coulter L, Everest DJ, Hill JE. Characterization of a novel poxvirus in a North American red squirrel (Tamiasciurus hudsonicus). J Wildl Dis. 2013;49:173–9. http://dx.doi.org/10.7589/2012-02-054
